# Transport of Carbamazepine, Ciprofloxacin and Sulfamethoxazole in Activated Carbon: Solubility and Relationships between Structure and Diffusional Parameters

**DOI:** 10.3390/molecules26237318

**Published:** 2021-12-02

**Authors:** Mohamed Bizi, Fatima-Ezzahra EL Bachra

**Affiliations:** BRGM, Water, Environment, Processes Development & Analysis Division 3, Avenue C. Guillemin, CEDEX 2, 45060 Orléans, France; elbachra.fatimaezzahra@gmail.com

**Keywords:** carbamazepine, ciprofloxacin, sulfamethoxazole, diffusion, drinking water, sorption kinetics

## Abstract

The transport of carbamazepine, ciprofloxacin and sulfamethoxazole in the different pores of activated carbon in an aqueous solution is a dynamic process that is entirely dependent on the intrinsic parameters of these molecules and of the adsorbent. The macroscopic processes that take place are analyzed by interfacial diffusion and reaction models. Modeling of the experimental kinetic curves obtained following batch treatment of each solute at 2 µg/L in tap water showed (i) that the transport and sorption rates were controlled by external diffusion and intraparticle diffusion and (ii) that the effective diffusion coefficient for each solute, with the surface and pore diffusion coefficients, were linked by a linear relationship. A statistical analysis of the experimental data established correlations between the diffusional parameters and some geometrical parameters of these three molecules. Given the major discontinuities observed in the adsorption kinetics, the modeling of the experimental data required the use of traditional kinetic models, as well as a new kinetic model composed of the pseudo first or second order model and a sigmoidal expression. The predictions of this model were excellent. The solubility of each molecule below 60 °C was formulated by an empirical expression.

## 1. Introduction

Carbamazepine (CBZ), Ciprofloxacin (CIP) and Sulfamethoxazole (SMX) are among the residues of pharmaceutically active compounds (PhACs) frequently found in various aquatic compartments. Various studies have confirmed their presence in certain surface waters, soils, sediments and groundwater [1,2,3,4,5,6]. A non-negligible quantity of these active ingredients and their metabolites is excreted, mainly in urine, and enters wastewater in urban areas or is directly released into the environment (in the case of livestock farms). Wastewater treatment plants (WWTPs) have been identified as the principal pathway for the discharge of pharmaceutical residues into the natural environment. Their efficient treatment of pharmaceutical residues depends on the physicochemical properties of the molecules and the characteristics of the treatment plants themselves (e.g., processes, hydrodynamic conditions, residence times, temperatures). The rates of abatement vary, both within a given treatment plant and from one plant to another. The volume of the contaminant load also depends on the operating conditions in the water treatment plant (e.g., plant shut-down, direct discharge of effluents during extreme rainfall events). The fate of pharmaceutical residues in the environment is determined in part by the physical and physicochemical characteristics of each molecule (such as their size, volume, molecular mass, solubility in water, dissociation constants, octanol-water distribution coefficient (D_ow_)). Their fate is also partly determined by environmental conditions: temperature, pH, ionic strength, presence of other chemical species, organic matter, matter in suspension, hydrodynamic conditions, hydraulic residence time, erosion, etc. The combination of these factors dictates the stability of the molecules in the environment. Although various advanced water treatment technologies exist, due to certain economic and technical constraints, the treatment of pharmaceutical compounds by adsorption is the preferred option in drinking water production and tertiary wastewater treatment. For this purpose, activated carbon is the most commonly used adsorbent at an industrial scale. This product has several advantages for this application: a high specific surface area with a good pore size distribution, attrition resistance, chemical stability both in acidic and basic conditions, thermal stability, surface chemistry very conducive to the adsorption of organic pollutants, a variety of forms (powder, granules, fibers, etc.) and a relatively low production cost. CBZ, CIP and SMX were chosen for this work because of their high consumption, occurrence in the environment and in sewage treatment plant, their high persistence and resistance to water treatment processes.

Carbamazepine, a neutral heterocyclic anticonvulsant, is a highly persistent substance [7,8,9]. It is neither degraded nor retained in wastewater treatment plants. In certain cases, its concentration can reach up to 6 µg/L in WWTP effluents [10], 8 µg/L in surface waters [3] and a few nanograms in drinking water [11]. It is considered a marker of the presence of wastewater in the aquatic environment and presents a potential risk factor for drinking water supplies. This molecule shows a high bioconcentration capacity within living organisms [12]. The half-life of CBZ in surface waters is between 60 and 100 days [13]. Based on the EC50 (half maximal effective concentration) provided in the literature, this compound could be considered highly toxic for algae, bacteria and invertebrates. A recent study conducted on the fate of CBZ in agricultural soils irrigated with wastewater from WWTPs showed that the sorption of this molecule is mainly governed by the level of organic matter in the soil [14]. Furthermore, CBZ can accumulate in certain plants (wheat, tomatoes, etc.) [15].

Ciprofloxacin (CIP), which belongs to the group of fluoroquinolones, is of particular interest because of its omnipresence in hospital effluents, wastewaters and surface waters at concentrations ranging from ng to mg/L. Near certain pharmaceutical factories, it can reach concentrations of up to 30 mg/L [16,17]. This antibiotic is also found in water intended for human consumption. Like most antibiotics, CIP has been shown to be relatively resistant to biodegradation and genotoxic for aquatic organisms [18]. Their presence in water, even at low concentrations, can promote the appearance of resistance in pathogenic bacteria [19] and can trigger inhibition in activated sludge plants. 

Sulfamethoxazole (SMX), an antibiotic for human and veterinary use, figures among the most commonly found residues in different aquatic compartments [3,6]. After being consumed, this antibiotic is metabolized in proportions not exceeding 70% [20]. A non-negligible quantity of this active ingredient and its metabolites is therefore excreted, mainly in urine, and enters wastewater in urban areas, or is directly released into the environment in the case of livestock farms. Its concentration can range from 10 to 1500 ng/L in WWTP effluents, from 0.2 to 1100 ng/L in groundwater, and from around 100 pg/L to a few ng/L in water intended for human consumption [4,5,6,21,22]. Heather E. Gall et al. [23] found 26 groundwater sites in the US (West Branch of the Susquehanna River basin) with SMX concentrations ranging between 0.1 and 32 µg/L, with an average of 17.1 µg/L. It was among the most prevalent antimicrobial contaminants detected in a nationwide groundwater survey conducted by the United States Geological Survey [24]. SMX can accumulate in certain plants (wheat, tomatoes, spinach) [25].

In our previous studies [26,27], we proposed the elimination of these molecules using activated carbon. The majority of the information given in these studies mainly concerned the choice of adsorbent and the specificities of the adsorption isotherms of molecules. It was then deemed useful to study the diffusion of these molecules through the different pores of this adsorbent in relation to the intrinsic properties of these molecules. To do so, it was necessary (i) to understand the sorption kinetics of these three molecules and the modeling of their diffusion and (ii) to find a direct relationship between the geometric dimensions of each molecule and its diffusivity in the pores of the adsorbent. It was also important to clearly define lipophilicity as a function of pH and the solubility of these molecules. Furthermore, examining very low quantities of contaminant in tap water was an original approach which tended toward realistic environmental situations. Certain characteristics of these molecules were more accurately defined in this study.

## 2. Results and Discussion

### 2.1. Characterization of PhACs 

The main characteristics of PhACs used in this study are given in Table 1.

**Figure 1:**
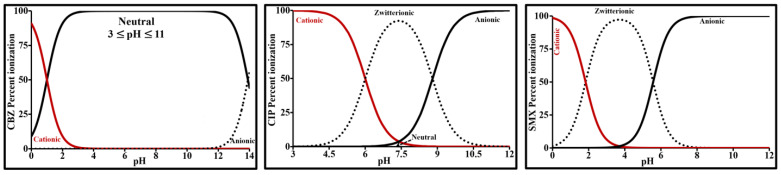
Speciation as a function of pH in aqueous solution.

Carbamazepine is a neutral, tricyclic, lipophilic compound that ends with a carboxamide group composed of a carbonyl group (-C=O) and a hydrophilic amide group (-NH_2_). The pKa values for this molecule are situated at each end of the usual pH range (Table 1). pK_a1_ and pK_a2_ correspond respectively to the protonation (RCONH_3_^+^) and deprotonation (RCONH^-^) equilibria of the amide group. Its speciation diagram (Figure 1) in pure water as a function of pH and its dissociation constants, established based on the Henderson-Hasselbalch method and Equation (1), shows that this molecule is neutral between pH 3 and 11.

(1) $$\mathrm{PI}\;=\;\frac{100}{1\;+\;10^{x\left(\mathrm{pH}-{\mathrm{pK}}_{a}\right)}}$$

where PI is the ionization percent. x = −1 and pk_a_ = pk_a1_ if acid drug or x = 1 and pk_a_ = pk_a2_ if basic drug.

CIP, a fluoroquinolone antibacterial agent, contains a secondary alkylamine, two tertiary arylamines (aniline-like amines), and a carboxylic acid. It has one fluorine, which is conjugated to the carboxylic acid functional group. With these multiple functional groups, CIP possess both acidic and basic characteristics. It is an amphoteric zwitterionic compound with acid dissociation constants of 6.0 ± 0.1 (pk_a1_) and 8.8 ± 0.1 (pk_a2_). As shown in Figure 1, it is primarily cationic below pK_a1_, anionic above pK_a2_, and zwitterionic/neutral between pk_a1_ and pk_a2_. pK_a1_ corresponds to the dissociation of the carboxylic acid and pKa2 to the protonation of the piperazinyl ring N atom (NH_2_^+^) [38,39]. Its isoelectric point (IEP = (pK_a1_ + pK_a2_)/2) is about 7.4.

SMX is a sulfonamide composed of a sulfonyl group located between an amine group and an aniline group. It has amphoteric properties with acido-basic characteristics. More specifically, it contains a basic amine group (-NH_2_) and an acidic amide group (-NH-). The amine group is able to accept a proton, while the amide group is able to donate a proton under specific pH conditions (Figure 1). This pharmaceutical compound is zwitterionic between its two pKa values, practically cationic below pK_a1_ and anionic above pK_a2_. It is completely anionic above pH 7.5. Its isoelectric point is about 4.

At pH 8, CBZ is totally neutral; CIP 1% neutral, 85% zwitterionic and 14% anionic; SMX is 100% anionic.

The physicochemical properties of CBZ and SMX mean that it is difficult to reduce their presence in soils. CBZ is neutral and SMX is anionic in alkaline water (WWTP and surface waters); both have low biodegradability, making them persistent and mobile in soils. At alkaline pH, electrostatic repulsion occurs between SMX and the main components of natural soil. CBZ shows no affinity for natural minerals. At pH 8, CIP can have a certain adsorption affinity for natural minerals and in particular for smectites. 

The values for the physical and physicochemical properties of these 3 pharmaceutical products (such as molecular mass, size, pH, log P, log D_ow_, and solubility) were used to assess and predict the probable sorption behavior of these 3 compounds. Their lipophilicity is represented by the descriptors log P and log D_ow_ (also known as Log K_ow_). The distribution coefficient D_ow_ can be used in the case of ionizable molecules; otherwise, it is identical to the partition coefficient P. The latter, given in Equation (2), measures the differential solubility of a single species (the compound) in two immiscible solvents: octanol and water. The distribution coefficient (Equation (3)), on the other hand, measures the differential solubilities of all species present in the two solvents (all forms, whether ionic or otherwise). P does not take into account the specific concentrations developed in relation to the pH. It can therefore lead to incomplete or incorrect interpretations in the case of ionizable compounds.

(2) $$P\;=\;\frac{{\left[\mathrm{Compund}\right]}_{\mathrm{octanol}}}{{\left[\mathrm{Compund}\right]}_{\mathrm{water}}}$$

(3) $$D_{\mathrm{ow}}\;=\;\frac{\sum {\left[\mathrm{Species}\right]}_{\mathrm{octanol}}}{\sum {\left[\mathrm{Species}\right]}_{\mathrm{water}}}$$

For CIP and SMX, which are ionizable solutes according to pH, the distribution coefficient D_ow_ is the appropriate descriptor as it represents the differential solubility measurement as a function of the pH of all the species in the octanol-water system. It is determined by the following relationship given for diprotic ampholytes [40]:

For diprotic ampholyte:

(4) $${\mathrm{Log}\;D}_{\mathrm{pH}}\;=\;{\mathrm{Log}\;P}^{\mathrm{IEP}}\;-\;\mathrm{Log}\left(1\;+\;10^{\left({\mathrm{pK}}_{a1}-\mathrm{pH}\right)}\right)\;+\;10^{\left(\mathrm{pH}-{\mathrm{pK}}_{a2}\right)}$$

**Figure 2:**
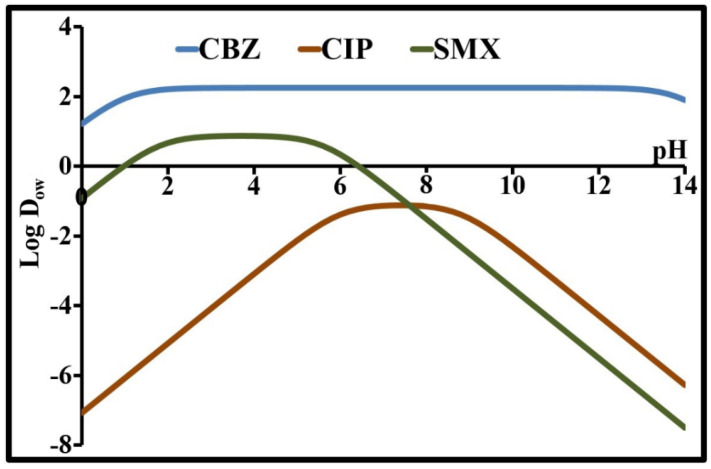
Lipophilicity-pH profiles of CBZ, CIP and SMX.

The pH dependency of Log D_ow_ is presented in Figure 2. The profiles of these 3 compounds show that ionization greatly affects octanol-water partition and that their lipophilicity cannot be simplified into a constant. The presence of a plateau for Log D_ow_ indicates the pH range for which the compound shows overall electrical neutrality due to a lack of charge or the existence of ion-pair partition. At the plateaus, lipophilicity is at its highest. The isoelectric points of CIP and SMX are located on these plateaus and the solubilities of these compounds at these points are at their lowest.

For CBZ, a neutral species, P and D_ow_ are identical and are equal to 2.25 in the pH range from 3 to 11. This value shows that this compound is preferably associated with a lipid phase. The negative Log D_8_ values for CIP and SMX suggest that these compounds would be more likely to have a high solubility in water and a low lipophilicity. These two compounds are more likely to be found in the water than in the organic matter of surface waters. Inversely, CBZ shows the opposite behavior.

Based on a few numerical values from experimental data taken from the literature [41,42,43], a simple expression was proposed to determine the solubility of these 3 molecules in pure water (Milli-Q water) at temperatures below 60 °C:

(5) $$S\;=\;P_{1}T\;+\;P_{2}T^{P_{3}}$$

The values of the Pi parameters are given in Table 1. T is in °C and the solubility S is in mg/L.

**Figure 3:**
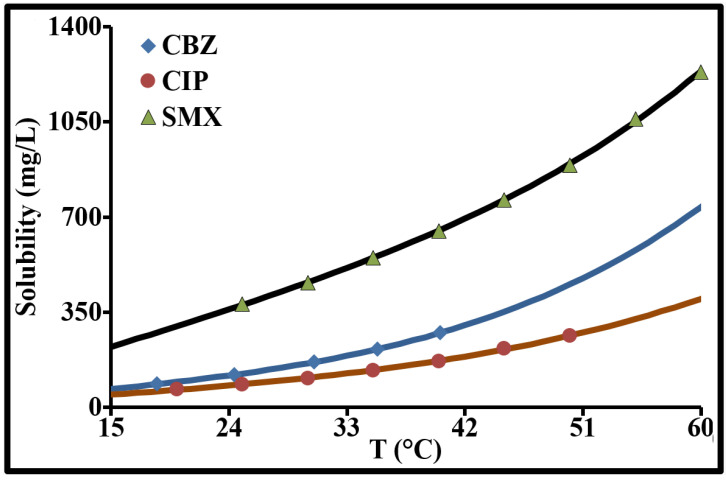
Solubilities of CBZ, CIP and SMX in water as function of temperature.

As indicated in Figure 2 and Figure 3, there is no direct correlation between the solubility and the distribution coefficient. The relationship between these two parameters can be reinforced by integrating other physicochemical parameters. Indeed, a compound’s solubility depends not only on its lipophilicity, the pH, pressure and temperature, but also on its molecular mass, molar volume, hydrogen bonds, ionizability, etc.

### 2.2. Activated Carbon Characterization

**Figure 4:**
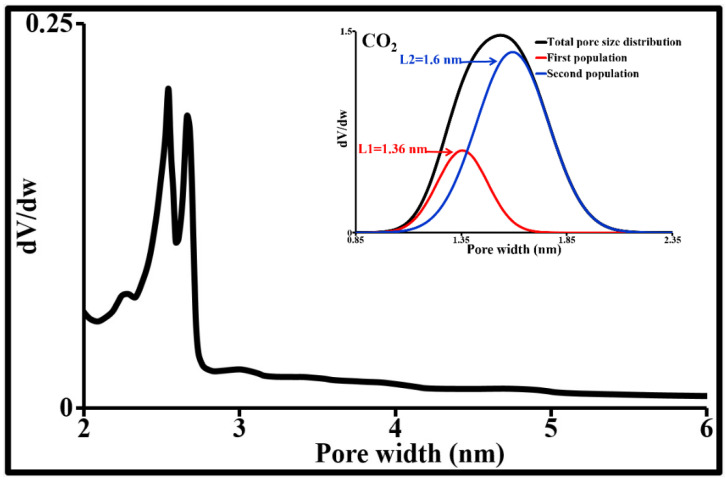
Pore size distribution obtained by N_2_ desorption at 77 K and CO_2_ adsorption at 273 K.

The textural properties of an adsorbent affect the adsorption equilibria. Knowledge of these properties is essential to more accurately interpret the adsorption mechanisms of all molecules, whether organic or otherwise. Textural analysis of the activated carbon powder (ACP) was therefore performed by N_2_ adsorption/desorption, CO_2_ adsorption and particle size measurement. Its main texture parameters are provided in Table 2. This activated carbon has a particle size of less than 120 µm and an average diameter of around 24 ± 1 µm. 7.5% by mass of its particle size distribution is below 2 µm and 56% is below 20 µm. It is also characterized by a large specific surface area, as well as a polydisperse pore size distribution, with 95% below 3 nm and 64% below 2 nm (maximum micropore size). Virtually all of its mesopores are between 2 and 6 nm. The macropore population (>50 nm) is negligible (Figure 4). These meso- and macropores can contribute to the transport of micropollutants by diffusion of the liquid phase into each particle. Macropores constitute pathways to the internal surface of the activated carbon. These pores govern diffusion rates, which in turn limit the solute adsorption rate. The microporous space represents 53% of the total porosity of ACP. It increases the sorption capacity. Furthermore, the overlapping force fields generated by the micropore walls lead to an increase in the adsorption potential within these cavities. The microporosity in this activated carbon is predominant. The minimum micropore size for this activated carbon (0.95 nm) is accessible to CBZ and SMX (width and height < 0.6 nm). CIP can reach pores larger than 1 nm. The specific surface areas of its micropores, mesopores and macropores represent respectively 73%, 21% and 6% of the total specific surface area according to the t-plot model. N_2_ and CO_2_ give virtually the same micropore surface areas and volumes.

Activated carbons have surface functional groups, the nature of which are dependent on the origin and chemical composition of their raw material as well as their preparation and activation method. These functional groups can be considered acidic or basic sites promoting ion adsorption. The most commonly found acidic sites at the surface of activated carbon are carboxyl (Ar-COOH), phenol (Ar-OH), carbonyl (Ar-C=O), anhydride (Ar-(C=O-O-O=C), ether (Ar(-O-)Ar’), quinone (O=Ar=O) and lactone groups (Ar-C=OO-Ar’). Basic sites can be associated with two types of structures: (i) chromene and pyrene; and (ii) Lewis structures associated with π electron-rich regions situated on basal planes [44,45,46]. Furthermore, part of the basicity could also be attributed to the intrinsic properties of the ash [47]. The identification and quantities of these functional groups, determined using the Boehm method, are provided in Table 3.

The sites revealed by this quantification method were present in very significant proportions, with the exception of lactones. Anhydride, carboxyl, lactone and phenol constituted the source of acidity in the surface of this carbon, while carbonyl was associated with basic groups. The phenol and carbonyl densities were almost 2 to 3 times higher than those of carboxyls and anhydrides. These functions make the carbon surface more polar and increase its affinity for water through the formation of hydrogen bonds. There were slightly more electron donors than acceptors. The concentrations of acidic and basic sites were 1.5 and 1.3 µmol/m^2^ respectively. All these functional groups determined the charge, hygrophylicity, hydrophobicity and density of the delocalized electrons π, and could explain the reactivity and differences in the activity of the activated carbon in relation to various substances. The hygrophylicity/hydrophobicity relationship was directly linked to the relationship between acidic and basic functions. According to the values obtained (1.5/1.3), Norit ACP was relatively hydrophilic. Its acidic nature should promote cation adsorption. These different chemical characteristics were corroborated by the point of zero charge (PZC) and isoelectric point (IEP) values, respectively 6 and 3.6 ± 0.1. The PZC is the pH that separates the protonation phase from the deprotonation phase of amphoteric functional groups. At this pH, the proton charge density is nil. In addition to OH^-^ and H^+^ ions which contribute to acid-base reactions with the surface hydroxyl groups, other ionic species are generally present at the solid/liquid interface and can lead to specific adsorption. The IEP is the pH at which the concentration of positive species at the surface is equal to the concentration of negative species. At this point, the total surface charge is nil. When IEP < PZC, this clearly indicates the presence of anionic species at the surface. Where pH < 3.6, the surface is positive; between 3.6 and 6, positive and negative charges are both present; above pH 6, the surface is entirely negatively charged. At tap water pH, the activated carbon Norit SA Super has a negative surface charge, CBZ is neutral and SMX is negative. If we exclude the cations present in tap water, the electrostatic interaction between SMX and ACP is completely repulsive. No electrostatic interaction can be produced between CBZ and ACP. However, at this pH, due to its zwitterionic state, CIP can be adsorbed under the effect of attractive electrostatic interaction.

### 2.3. Sorption Kinetics of the Three Target Contaminants

#### 2.3.1. Applied Sorption Models

The analysis of the sorption kinetics of each target contaminant provided information that may be used to map the mechanisms required to interpret the phenomena at play and to design response tools to optimize collection of the contaminant. In the case of a porous sorbent such as activated carbon, this analysis constitutes a key element in determining (i) the rate at which molecules in the solute are able to reach active sorption sites; (ii) the rate-limiting steps in molecule transport within the adsorbent and (iii) the reactivity and adsorption capacity of the adsorbent. In the given physicochemical conditions, the adsorption rate is controlled by phenomena governing the transport in and out of the adsorbent’s pores. The transport steps, whether separate or combined, form multiple diffusion resistances which limit the rate at which the contaminant can reach the adsorption sites. Sorption occurs in four successive steps [48,49]: (1) diffusion of the adsorbate from the liquid phase towards the diffusive boundary layer of the liquid surrounding the adsorbant particle; (2) diffusion through this layer to the outer surface of the adsorbant (diffusion of the film or external diffusion); (3) diffusion through the inner pores of the adsorbant particles (intraparticle diffusion or internal diffusion); and (4) energetic interaction between the adsorbate and the adsorption sites (adsorption to the surface of the pores). In general, the approaches used to study and model these phenomena build on Fick’s first law and Fick’s second law [50]. Fick’s first law determines the influence of the concentration gradient of the diffusing substance on the diffusive flux:

(6) $$\vec{J}\;=\;-D\vec{\mathrm{grad}}C$$

Taking into account the law of conservation of mass, Fick’s second law, a fundamental differential equation of diffusion, is given by the following relationship:

(7) $$\frac{\partial C}{\partial t}\;=\;\mathrm{Ddiv}\vec{\mathrm{grad}}C$$

where J is the rate of transfer per unit area of section (flux); C is the concentration of the diffusing species; t is the time and D is the diffusion coefficient (also called diffusivity). Based on these two laws, the relationships between the concentration of the reaction medium, time and diffusivity can be expressed. In spherical coordinates and for radial diffusion and a diffusivity independent of concentration, Fick’s second law is expressed:

(8) $$\frac{\partial C}{\partial t}\;=\;\frac{D}{r^{2}}\frac{\partial }{\partial r}\left(r^{2}\frac{\partial C}{\partial r}\right)$$

The equivalent of this relationship for relative adsorption is:

(9) $$\frac{\partial q}{\partial t}\;=\;-\frac{D}{r^{2}}\frac{\partial }{\partial r}\left(r^{2}\frac{\partial q}{\partial r}\right)$$

where

(10) $$q_{t}\;=\;\frac{V}{m}\left(C_{0}\;-\;C_{t}\right)$$

where q_t_ is the amount of solute sorbed at time t; V is the volume of solution; m is the mass of dry adsorbent; C_0_ is the initial solute concentration; C_t_ is the concentration of the solute at time t; r is the radial distance from the center of the sphere. 

The analytical solutions for these two relationships depend on the systems studied, and on the boundary and initial conditions. 

##### External Diffusion

Mass transfer of the solute through the film of liquid surrounding the adsorbent particles is assumed to be controlled by a perpendicular flow at the outer surface of the solid, and variation in concentration over time is related to the fluid-particle mass transfer coefficient by the following equation [50,51]:

(11) $$V\frac{\mathrm{dC}}{\mathrm{dt}}\;=\;-{\mathrm{mSS}}_{\mathrm{ex}}k_{f}\left(C\;-\;C_{S}\right)$$

$$C\;=\;C_{0}\;\mathrm{at}\;t\;=\;0$$

where V is the solution volume (m^3^), C and C_S_ are the solute concentration in solution and at the solid-liquid interface, respectively (mg/L), t is the time (s), m is the adsorbent mass (g), SS_ex_ is the external specific surface area of the adsorbent (m^2^/g), k**_f_** is the external mass transfer coefficient (m/s). 

The parameter k**_f_** represents the diffusion rate of molecules through the diffusive layer of liquid film. The thickness of the boundary layer depends on the hydrodynamic conditions of the fluid flow around the adsorbent particle.

##### Intraparticle Diffusion

During an isothermal, isobaric process in the presence of identical spherical adsorbent particles with radius R in a perfectly agitated solution—and if the initial concentration inside the particles is nil and that at the surface is a function of time (f(t))—the solution to Equation (4) is determined as follows [50,52]:

(12) $$q\left(r,t\right)\;=\;\frac{2}{\mathrm{Rr}}\overset{\infty }{\underset{i=1}{\sum }}{\left(-1\right)}^{i+1}\;i\;\pi \;D\;\exp\left(-{\left(\frac{i\pi }{R}\right)}^{2}\mathrm{Dt}\right)\sin\left(\frac{i\pi r}{R}\right)\int_{0}^{t}\;\exp\left({\left(\frac{i\pi }{R}\right)}^{2}D\lambda \right)q_{s}\left(\lambda \right)d\lambda$$

The initial and boundary conditions are

$$\mathrm{at}\;t\;=\;0,\;0\;\leq r\;\leq R:\;C\;=\;C_{0}{\;\mathrm{and}\;q}_{0}\;=\;0\;\mathrm{at}\;t\geq 0,\;r\;=\;0:\;\frac{\partial q\left(0,\;t\right)}{\partial r}\;=\;0\;\mathrm{at}\;t\geq 0,r\;=\;R:\;q\;=\;q_{S}$$

In application, there exist several diffusion prediction models of varying complexity, using specific hypotheses aimed at reducing the complexity of this analytical solution. One commonly used model is the Homogeneous Surface Diffusion Model (HSDM). This model builds on the fact that the overall adsorption reaction is kinetically limited by external diffusion and homogeneous diffusion in the adsorbent. Adsorbent particles are assumed to be a solid, homogeneous sphere in which the adsorbate is transported towards the center of the particle by surface diffusion with constant diffusivity. It is assumed that external mass transfer is governed by a linear driving force and that there is a continuity between external and internal mass transfer [53,54,55]. These hypotheses are accepted, as a first approximation, in the case of the activated carbon Norit SA Super, in order to determine the surface diffusion coefficients and to model the adsorption kinetics of CBZ, CIP and SMX according to the HSDM. As previously indicated, 95% of the pores of this activated carbon had pore opening widths of less than 3 nm and 64% had less than 2 nm. The walls of these porous slits were, therefore, quite close to each other. This proximity could lead to overlapping intense force fields with an increase in the adsorption potential and surface diffusion. Adsorbates between 0.5 and 1.3 nm in size will always be very close to the walls and cannot escape their influence. They will mainly be transported by surface diffusion. Several numerical methods exist to apply the HSDM. Its mathematical formulation involves nonlinear equations which include physical and kinetic parameters and assumes that adsorption is described by the Freundlich adsorption isotherm. In this study, the approach developed by Li Ding was followed and the author’s MATLAB code was applied [56]. This code was formulated based on the following analytical equations:

(13) $$q_{t}\;=\;\frac{1}{\frac{4}{3}{\pi R}^{3}}\int \int \int q\left(r,t\right)\;\mathrm{dV}\;=\;\frac{3}{R^{3}}\overset{R}{\underset{0}{\int }}q\left(r,t\right)r^{2}\;\mathrm{dr}$$

(14) $$q_{t}\left(t\right)\;=\;\frac{6D_{S}}{R^{2}}\overset{\infty }{\underset{i\;=\;1}{\sum }}\exp\left(-{\left(\frac{i\pi }{R}\right)}^{2}D_{s}t\right)\int_{0}^{t}\;\exp\left({\left(\frac{i\pi }{R}\right)}^{2}D_{s}\lambda \right)q_{s}\left(\lambda \right)d\lambda$$

(15) $$q_{s}\left(\lambda \right)\;=\;K_{F}C{\left(\lambda \right)}^{1/n}$$

$$\mathrm{For}\;t\geq 0,\;q\;=\;q_{S}\left(t\right)\;\mathrm{at}\;r\;=\;R\;\mathrm{and}\;\frac{\partial q}{\partial t}\;=\;0\;\mathrm{at}\;r\;=\;0$$

where q_t_ is the amount of solute sorbed at time t; r is the radial distance from the center of the particle; R is the mean radius of the porous particles; and q_S_ is the adsorbate load at liquid/solid interface. Additionally, q_S_ is related to the bulk solution concentration by the isotherm equilibrium, which in our case is the Freundlich isotherm (Equation (15)). K_F_ is the Freundlich isotherm constant; 1/n is the Freundlich isotherm intensity constant (dimensionless); D_S_ is the surface diffusion coefficient.

The adsorbent is assumed to be unused; the kinetics are generated by homogeneous surface diffusion and the adsorption equilibrium is described by the Freundlich adsorption isotherm. Thanks to the numerical resolution of these equations, it is possible to obtain standardized and nonstandardized aqueous concentrations as a function of time and the surface diffusion coefficient. The model also calculates the difference between the model prediction and the experimental data.

The rate-limiting steps (external and/or internal) were demonstrated using the Weber–Morris intraparticle diffusion model [48]. This model divides changes in equilibrium that may occur during sorption according to a proportional relationship between the quantity adsorbed and the square root of the time shown during short durations:

(16) $$q_{t}\;=\;k_{i}\sqrt{t}\;+\;I$$

where q_t_ is the relative quantity sorbed during time t (mg/g), k_i_, the slope of the line (mg/g min^0.5^), is the intraparticle diffusion constant and I, the y-intercept (mg/g), is a constant that provides information on (i) the effect of the boundary layer and (ii) the relative importance of the two solute transport mechanisms (mg/g). If I = 0, intraparticle diffusion is considered to be the rate-limiting step, whereas if I > 0, external mass transfer and intraparticle diffusion are considered to be the rate-limiting steps.

To study the 4th step, which corresponds to the adsorption reactions, the relevance of the pseudo-first-order and pseudo-second-order kinetic models, and many others models, were determined. The nonlinear forms of the equations were used to interpret the experimental results. In order to quantitatively compare the applicability of different kinetic models in fitting the data, a normalized standard deviation, ∆q, was also calculated as follows:

(17) $$\Delta q\left(\%\right)\;=\;100\sqrt{\frac{1}{n\;-\;p}\sum {\left(\frac{q_{t,\exp}-q_{t,\mathrm{cal}}}{q_{t,\exp}}\right)}^{2}}$$

where n is the number of experimental data; p is number of free parameters of the model; q_t,exp_ and q_t,cal_ are the experimental values and the values calculated by the model, respectively. 

The appropriate model to describe the sorption kinetics of each system was determined based on the comparison of the coefficient of determination R^2^ and the normalized standard deviation ∆q (%). The determination of R^2^ alone was insufficient to decide among the kinetic models.

#### 2.3.2. Impact of the Dosage of ACP on the Elimination Rate of Each Pollutant

**Figure 5:**
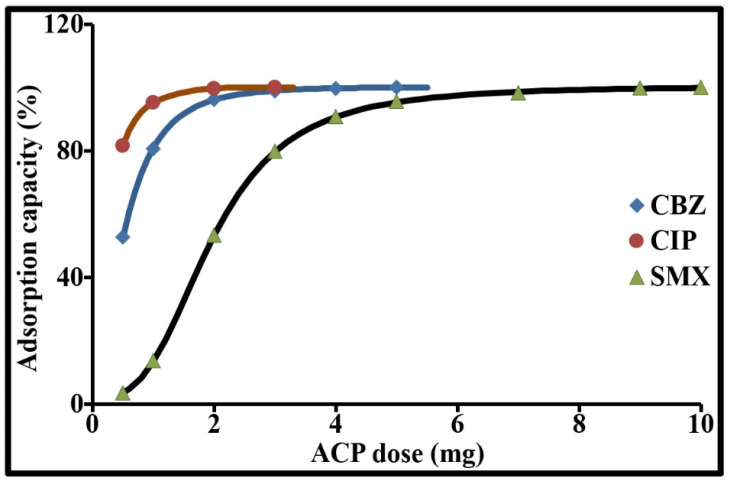
Effect of adsorbent dosage on the adsorption of CBZ, CIP and SMX (drinking water, pH 8.1 ± 1, 20 ± 1 °C, 2 µg/L of pollutant, Equilibrium time 4 h).

The impact of the dosage of ACP on the elimination rate of each pollutant initially introduced at a concentration of 2 µg/L is shown in Figure 5. This figure demonstrates that elimination increased as the dosage of adsorbent increased. The dosages required to eliminate 99 ± 1% were: 5 mg/L for CBZ, 2.5 mg/L for CIP and 10 mg/L for SMX. Beyond these values, adsorption stabilized at around 100% in equilibrium conditions at 20 ± 1 °C, pH 8.1 ± 1, for 4 h. The increase in adsorption with increased dosage is attributed to the large specific surface area, the texture of the adsorbent, and the availability of a large number of active adsorption sites. These optimal dosages were used to determine the sorption kinetics. The isotherms obtained from these data were simulated using the Freundlich model (Equation (15)) which aims to extract the Freundlich intensity coefficient 1/n required to apply the HSDM. With this model, K_F_ and 1/n are constants associated with the Freundlich adsorption capacity and adsorption intensity. The value of the coefficient 1/n is directly related to the shape of the isotherm and the energetic heterogeneity of the adsorbent. For a favorable adsorption process, this coefficient is less than 1. When 1/n is equal to 1, the isotherm is linear; between 1 and 0, the isotherm becomes increasingly concave. The values of this coefficient for CBZ, CIP and SMX were 0.31, 0.40 and 0.25, respectively. The fact that they were all below 0.5 showed that the adsorption of these pollutants by activated carbon was very favorable. The experimental data were fitted with a coefficient of determination R^2^ of 0.99 and a normalized standard deviation (Equation (17)) of 1.5% for CBZ, 3.1% for CIP and 2.5% for SMX. For a pollutant concentration of 2 µg/L, this model is compatible with the heterogeneous surface of this adsorbent.

#### 2.3.3. Degradation Kinetics

Adsorption kinetics occur in a closed environment containing an initially defined mass of solid, volume of solution and quantity of pharmaceutical compound. Every time step corresponds to a suspension and a data analysis (one bottle per time step).

**Figure 6:**
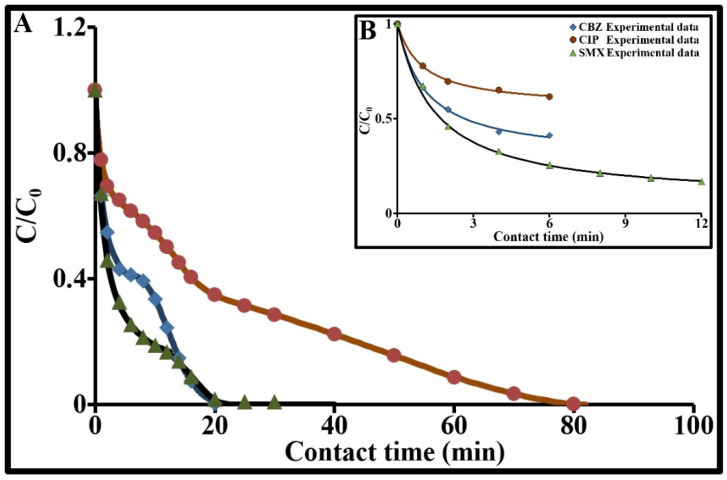
Dimensionless concentration as a function of time for CBZ, CIP and SMX adsorption by ACP in drinking water at 293 K (Values given at ±2 ng L-1 for each solute; (**A**) 0 to 100 min; (**B**) 0 to 12 min).

Figure 6 illustrates the influence of contact time on the individual sorption processes of the three molecules on Norit ACP. In accordance with the dosage choices, the kinetic curves were marked by a continual drop in residual concentrations until complete elimination of each molecule in the solution is achieved. The relative profiles of these kinetics indicated (i) rapid sorption of CBZ and SMX compared to CIP, and (ii) a rapid, sharp drop in the SMX concentration compared to CBZ during the first 13 min. After 13 min, elimination reached 85% for SMX, 80.5% for CBZ and 52% for CIP. Sorption at the surface and in the different porous compartments of the ACP will, of course, depend on the concentration gradient in the solution—and also on the intrinsic characteristics of the ACP and of each molecule. CIP, which is zwitterionic and has a greater molecular mass and larger geometric dimensions than CBZ and SMX, showed relatively slower kinetics. The inflection points observed on each curve corresponded to the beginning and end of the different sorption processes undergone by the contaminant. The first inflection point marked out a zone that characterized the initial sorption rate. This initial rapid phase lasted 6.4 min for both CBZ and CIP and 14.2 min for SMX. Following this phase, around 59% of CBZ, 39% of CIP and 86.5% of SMX was eliminated. This phase resulted from an initially high concentration gradient and varied from one product to another according to the molecular mass and the size and chemistry of each molecule (polarity, charge, etc.).

#### 2.3.4. Intraparticle Diffusion

**Figure 7:**
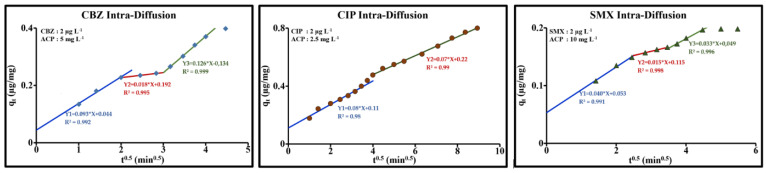
Weber-Morris plots for the sorption of CBZ, CIP and SMX by ACP Norit SA Super in drinking water (2 µg/L; ACP: 10 mg/L; pH = 8.1, T = 293 K).

The Weber–Morris intraparticle diffusion model [48] (Equation (16)) was used as an initial approach to describe the sorption processes and to determine, in particular, the rate-limiting steps for solute sorption. In all three cases, the plots for q_t_ over √t showed multiple, successive, linear sections (Figure 7). Slope k_i_ of each section is a diffusion rate parameter which characterizes the sorption kinetics in the region where diffusion in the pores is the rate-limiting step. As illustrated in Figure 7, the y-intercept I of each subject was greater than zero; external mass transfer and intraparticle diffusion were therefore considered sorption rate-limiting steps. The overall sorption rate will be controlled by the slowest step, which corresponds to the rate-limiting step. The value for this y-intercept also provides information on the effect of the boundary layer; the higher the I value, the greater the effect. The k_i_ and I values for each molecule are given in each relevant figure. In the operating conditions applied and at the scale of the values measured, this dynamic process can be divided into 4 steps in the case of CBZ and SMX, and two steps for CIP.

The first step (1st line) for the three molecules corresponds to diffusion in an almost entirely continuous medium formed by the outer surface and the macropores. The positive value for the y-intercept (I > 0) determined at t = 0 min suggests that a quantity of solute was present in the boundary layer of the activated carbon powder at t = 0 min, the time taken as the measurement start time. There was a delay between the real time taken by the molecules and the experimental time. This delay effect was not integrated in the Weber–Morris approximation. Whatever the case, the rate of this initial phase was very favorable for CBZ (neutral) and CIP (zwitterionic), and, as expected, the y-intercepts (intercept = I_i_) for the 3 molecules strongly correlated to their molecular weights (I = 6.83 × 10^−4^ × MW − 0.119, R^2^ = 0.998). The 3 other successive steps, in the case of CBZ and SMX, can characterize, in the following order: diffusion in the mesopores, diffusion in the micropores and, finally, an equilibrium phase during which the diffusion rate decreases in favor of stable sorption.

The transport rate was higher during the first step (external surface and macropores) than during the second step, attributed to the mesopores (k_i1_ > k_i2_). In the mesopores, the CBZ rate constant was far lower, dropping from 0.09 to around 0.02 mg/g min^0.5^, then suddenly rising by passing into the micropores (k_i3_ ≈ 0.13 mg/g min^0.5^). This increase was very probably due to far more dominant surface diffusion due to a narrowing of the walls and conducive reactivity of the surface functional groups. The negative value for the y-intercept I_3_ for micropores in the case of CBZ revealed a resistance to diffusion at the mesopore/micropore interface and the insufficient capacity of micropores to manage a relatively overloaded flow. It is possible that there may be backflow into the mesopores, which would lead to a decrease in the mesopore rate constant. This process is less marked than in the case of SMX. However, k_i_ drops from 0.04 to 0.01 then rises to 0.03 mg/g min^0.5^, and I drops from 0.05 to 0.01 then rises back up to 0.05 mg/g. In the case of CIP, the second step can be attributed to diffusion in the micropores. At the scale of the observation, diffusion in the mesopores was not easily observed. The effect of these cavities was indicated by a brief change of slope between 14 and 16 min. 

It follows that (i) the overall sorption rate will be controlled by the slowest step, which corresponds to the rate-limiting step (low k_i_); (ii) the transition pores (macro-mesopores) play a determining role in molecule transport to the micropores; (iii) according to the cavity dimensions, diffusion may be volume diffusion, surface diffusion or a combination of the two. The adsorption yield is dependent on the physical and physicochemical characteristics of the adsorbate, adsorbent, fluid phase and the operating and hydrodynamic conditions applied.

#### 2.3.5. External Diffusion

Mass transfer of the solute through the film of liquid surrounding the adsorbent particles is assumed to be controlled by molecular diffusion. The characteristics k_f_ and C_S_ can be determined by finding a numerical or analytical solution to Equation (11) by assuming that the concentrations in the solution and in the adsorbent are uniform and that the diffusion through the boundary layer is quasi-stationary. The analytical model often applied assumes that the surface concentration can be considered negligible (C_S_ = 0) during the initial moments of transfer. Hence, this equation is reduced to a simple expression used to estimate k_f_ from the initial slope of the Log C curve as a function of time [51]. In the absence of the second parameter, this method underestimates the k_f_ values. The values obtained for CBZ, CIP and SMX were 0.07, 0.09 and 0.04 cm/min, respectively. 

In this study, we proposed to maintain these two parameters in Equation (11) and to address the issue as follows: (1) fit the time evolution of C using Chase’s empirical equation [57] (Equation (18), Figure 6B) for the most appropriate time interval and, in particular, below the first inflection point (0 to 6 min for CBZ and CIP and 0 to 12 min for SMX); (2) calculate the derivative of the fitting data using a small time step (∆t = 0.02 min, for example); (3) plot the function −dC/dt as a function of time (f(C)); (4) determine, by linear regression, the slope and the y-intercept of the linear function −dC/dt = αC + β which represents the initial data; (5) extract k_f_ and C_s_ from the following relationships:

(18) $$\frac{C}{C_{0}}\;=\;1-a_{1}\left\{\frac{a_{2}\left[1\;-\;\exp\left(-2a_{3}t\right)\right]}{a_{4}-\exp\left(-2a_{3}t\right)}\right\}$$

(19) $$-\frac{\mathrm{dC}}{\mathrm{dt}}\;=\;\frac{{\mathrm{mSS}}_{\mathrm{ex}}}{V}k_{f}C\;-\;\frac{{\mathrm{mSS}}_{\mathrm{ex}}}{V}k_{f}C_{S}$$

(20) $$\alpha \;=\;\frac{{\mathrm{mSS}}_{\mathrm{ex}}k_{f}}{V}$$

(21) $$\beta \;=\;-\frac{{\mathrm{mSS}}_{\mathrm{ex}}k_{f}C_{S}}{V}$$

This method was performed using Origine software, although it could also be performed using MATLAB. The values for parameter a_i_ were not used in this paper and are therefore not provided. Chase’s model draws primarily on the second-order kinetic model and uses a single, global, kinetic constant k_CH_ which was included in parameter a_3_. This model showed excellent fit (Figure 6B) with a coefficient of determination R^2^ of 0.999 and a relative normalized standard deviation ∆C/C_0_ of 3.1% for CBZ, 1.1% for CIP and 1.7% for SMX.

In similar, homogeneous hydrodynamic conditions (viscosity, Reynolds number, local turbulence, dispersion, etc.) for the 3 solutes, the mass transfer coefficient for CIP through the liquid film is faster than for CBZ and SMX (Table 4). The differences observed between these coefficients were due in part to their geometry, their form factor, and their momentum. The first correlations took the form of direct relationships between the cross-sectional area (LxH) and Log k_f_:

(22) $${\mathrm{Logk}}_{f}\;=\;-2.726\;\times \;\left(L\;\times \;H\right)\;+\;0.955,{\;R}^{2}\;=\;0.996$$

Furthermore, the concentration at the outer surface was higher for CIP.

#### 2.3.6. Diffusion Prediction with HSDM Model

**Figure 8:**
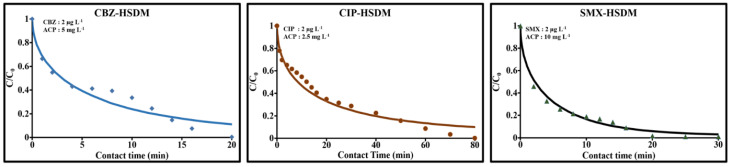
HSDM model prediction of surface diffusion coefficients of CBZ, CIP and SMX.

Based on the hypothesis of dominant, homogeneous surface diffusion, the homogeneous surface diffusion model (HSDM) was applied to the experimental sorption data for CBZ, CIP and SMX. As expected, the predicted curves presented in Figure 8 did not fit the experimental data of the last zones located at t > 14 min for CBZ and SMX and t > 50 min for CIP. These zones characterized continuous adsorption evolving towards an equilibrium plateau. Furthermore, the gap between the experimental values and the predicted curves in the 6 to 14 min zone could reveal the presence of volume diffusion that is not taken into account by the HSDM. The surface diffusion coefficients values D_S_ found were: 0.9 × 10^−10^ cm^2^/min for CIP, 3.4 × 10^−10^ cm^2^/min for CBZ and 12.2 × 10^−10^ cm^2^/min for SMX. As observed, the diffusivity of SMX was far higher than that of CBZ and CIP. For fixed geometric characteristics of the adsorbent, the gaps between these diffusivities were due to differences between the characteristics and the intrinsic properties of these molecules, as well as the reactivity of the ACP with each molecule. The correlations between the different parameters determined in this study and the identification of the influence of each of them on surface diffusivity were examined by correlation matrix analysis. This method provided a simple overview of objects considered according to all their known fields of variation. It could be used to sort the variables and reduce the number of parameters required to describe observations, minimizing information loss. The correlations, probability values and coefficients of determination are summarized in Table 5a–c.

The statistical study showed that Log D_S_ correlated to k_f_, C_S_, Log D_e_, and to the products of the height (H) by the length (L) or by the width (W) of each molecule. The strongest correlations were given for the LxH product and for Log C_S_. The correlation was positive when the two variables evolved in the same direction, and negative when the variables evolved in opposite directions. The calculation of probabilities clearly indicated that the probability of the correlation with LxH or with Log C_S_ occurring randomly with an Alpha significance level of 0.05 was practically nil (*p* ≤ 0.002). In addition, the coefficient of determination of these two correlations was equal to 1. In the same way, k_f_ and Log D_e_ were correlated to the geometric cross-sectional area of molecules, LxH. The quantity adsorbed at equilibrium (q_e_) was positively correlated to Log k_f_, and negatively correlated to LxH, Log D_S_ and Log D_e_. The correlations of the pairs (q_e_, LxH) and (q_e_, Log D_S_) can also be distinguished by R^2^ = 1 and *p* ≤ 0.005. Based on this analysis, and the experimental configuration of this research, it is highly probable that the main correlations are described by the following linear regressions

(23) $${\mathrm{LogD}}_{S}\;=\;+4.105\;\times \;\left(L\;\times \;H\right)\;-\;11.395,{\;R}^{2}\;=\;1$$

(24) $$q_{e}\;=\;-0.123\;\times \;{\mathrm{LogD}}_{S}\;-\;0.929,{\;R}^{2}\;=\;1$$

(25) $$q_{e}\;=\;-0.298\;\times \;{\mathrm{LogD}}_{e}\;-\;2.076,{\;R}^{2}\;=\;1$$

The molecular mass, boiling point and dimensions of the molecule are interdependent. The boiling point is directly related to the bond energies of the atoms that make up the molecule and to the interatomic distances.

#### 2.3.7. Effective Diffusivity Prediction

The effective diffusion coefficient D_e_ is an important parameter to describe solute transport in porous media. This parameter depends not only on the characteristics and intrinsic properties of the molecules carried by the fluid, but also on the properties of the surface and the geometric structure of the porous media: electric surface charge, hydrophobicity, porosity, tortuosity, constrictivity, etc. It is difficult to determine locally. However, its macroscopic value can be determined by means of certain hypotheses. In a homogeneous suspension composed of a solute and monodisperse spherical porous particles with a mean radius R and whose volume fraction occupied by the solute is negligible—as was the case in this study—in which the concentration of each contaminant is 2 µg/L, the effective diffusion coefficient can be calculated from the initial sorption data. During the first transport and adsorption step, and during the short time periods when q_t_/q_e_ < 0.3, Fick’s second law can be approximated as follows [50,58,59]:

(26) $$\frac{q_{t}}{q_{e}}\;=\;\frac{6}{R}\sqrt{\frac{D_{e}t}{\pi }}-\frac{3D_{e}t}{R^{2}}$$

q_t_ and q_e_ have been initially defined, D_e_ is the effective diffusion coefficient, t is time and R is the mean radius of the porous particles.

The fitting of the experimental data qt/qe as a function of t for q_t_/q_e_ < 0.3, by applying this relationship (y = at^0.5^ + bt) using the Origine software, gave an effective diffusion coefficient of around 1.7 × 10^−8^ cm^2^/min for CBZ, 1.1 × 10^−8^ cm^2^/min for CIP, and 3.1 × 10^−8^ cm^2^/min for SMX (±0.1 × 10^−8^ cm^2^/min for the 3 solutes). 

Unsurprisingly, and in agreement with the observations shown in Figure 5, the sorption of SMX, a fully anionic molecule at pH 8.2, was far faster than the other substances. The effective diffusion coefficient for this molecule was twice as high as that of CBZ and 3 times as high as that of CIP. These coefficients, as well as the surface diffusion coefficient obtained for the three molecules studied, were interdependent. Together, they form a linear function, as reported by Streider et al., [60] and Chen et al., [61]:

(27) $$D_{e}\;=\;17.3\;\times \;D_{S}\;+\;1.0\;\times \;10^{-8},{\;R}^{2}\;=\;0.99$$

The slope of this relationship depends on various factors, including the porosity, tortuosity, and average pore opening width. The y-intercept of this relationship represents the pore diffusion coefficient of the ACP, D_p_. The decrease in the diffusivity of the pores compared to the effective diffusivity is due to a diffusivity of the molecules on the surfaces of the pore walls. The adsorption process of these 3 contaminants is governed by diffusion within pores and in particular, surface diffusion coefficient.

#### 2.3.8. Individual Adsorption Kinetics of CBZ, CIP et SMX

**Figure 9:**
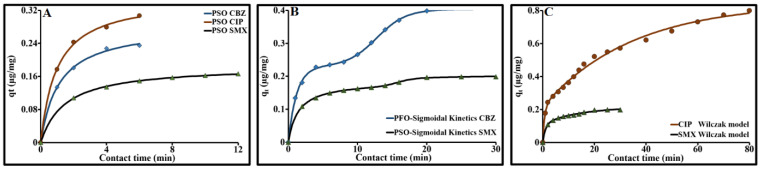
Modeling the adsorption kinetics of CBZ, CIP and SMX (**A**) PSO Fit; (**B**) PFO-Sigmoidal fit for CBZ, PSO-Sigmoidal fit for SMX; (**C**) Wilczak fit).

The purpose of studying adsorption kinetics is to determine the average adsorption rates, which in turn control the time required to reach adsorption equilibrium and to identify the dominant adsorption process. Several kinetic models have featured in the literature. In this study, the choice of kinetic models selected to analyze adsorption stemmed directly from the adsorption behavior in connection with the texture and functional groups which characterized the activated carbon Norit SA Super. Discontinuities appeared in the sorption kinetics of CBZ, CIP and SMX, marked by inflection points in the curves for the quantities adsorbed as a function of contact time (Figure 9). Each inflection point marked a rupture between two different sets of physical and physicochemical conditions. The kinetic models selected address the average overall behaviors. Sorption is governed by two processes: accumulation and rapid adsorption at the surface, followed by slower penetration. Inside the pores, adsorption and diffusion are intermingled. The slowing of sorption kinetics, illustrated by the evolution of q_t_ (Figure 9), occurs all the more rapidly when the ACP is loaded with solute. This phenomenon is characteristic of kinetics governed by concentration gradients.

Four kinetic models were applied to describe the behaviors observed: a pseudo-first-order reaction model (PFO, Equation (28)), a pseudo-second-order model (PSO, Equation (29)), a double exponential model (Equation (31)) and, finally, the fourth model that we propose in this paper: a new model formed by the arithmetical sum of the PFO or PSO model and a sigmoidal model (Equations (32) and (33)). These models are described by the following equations:

(28) $$q_{t}\;=\;q_{e}\left[1\;-\;\exp\left(-k_{1}t\right)\right]$$

(29) $$q_{t}\;=\;q_{e}\left[1\;-\;\frac{1}{1\;+\;q_{e}k_{2}t}\right]$$

(30) $$h\;=\;{\;k}_{2}q_{e}^{2}$$

(31) $$q_{t}\;=\;q_{0}\;+\;{\;q}_{r}\left[1\;-\;\exp\left(-k_{s}t\right)\right]\;+\;q_{s}\left[1\;-\;\exp\left(-k_{r}t\right)\right]$$

(32) $$q_{t}\;=\;q_{e}\;+\;q_{e}\left[1\;-\;\exp\left(-k_{1}t\right)\right]\;+\;\frac{\left(q_{0}\;-\;q_{e}\right)}{\left[1\;+\;\exp\frac{\left(t-t_{0}\right)}{\mathrm{dt}}\right]}$$

(33) $$q_{t}\;=\;q_{e}\;+\;q_{e}\left[1\;-\;\frac{1}{1\;+\;q_{e}k_{2}t}\right]\;+\;\frac{\left(q_{0}\;-\;q_{e}\right)}{\left[1\;+\;\exp\frac{\left(t\;-\;t_{0}\right)}{\mathrm{dt}}\right]}$$

where q_t_ is the amount of adsorbed solute at time t (mg/g), q_e_ is its value at equilibrium (mg/g), k_1_ is the pseudo-first order rate constant (1/min), k_2_ is the pseudo-second order kinetic rate constant (g/mg min), q_0_ is the amount of adsorbed solute at t = 0 min, q_r_ and q_s_ are the amount of adsorbed solute of the rapid and the slow step, respectively, k_s_ and k_r_ are rate parameters (1/min), t is time (min), t_0_ is the point of inflection, and dt is the width.

The kinetic data were fitted by nonlinear regression. This model was selected as it offered the best prediction of the experimental results for adsorption equilibrium. The compliance of the model was better when the coefficient of determination R^2^ was higher and the error function ∆q% was lowest. The kinetic parameters obtained from these models are presented in Table 5. Below, for the first discontinuity at around 6.4 min for CBZ and CIP and at 14 min for SMX, the PSO model was best suited. This prediction steers adsorption reactions towards governance of a chemical nature. This dynamic phenomenon is due to surface reactions generated by different types of compatibility between the surface of the activated carbon and these 3 molecules.

As shown in Figure 9, and transcribed numerically in Table 5, the adsorption reaction of ciprofloxacin is faster than those of CBZ and SMX. This increase in the initial rate h and the decrease in the pseudo-second-order kinetic constant k_2_ directly followed the increase in the contaminant mass in relation to the mass of activated carbon and that of the mass transfer coefficient k_f_. For a constant volume of suspension, the increase in the number of moles (concentration) triggered a rise in the pull force of the concentration gradient and, therefore, an increase in molecular diffusion and a consequent increase in the mass transfer coefficient k_f_.

Independently of the molecules’ specific characteristics, there was a linear relationship between the initial rates h and both the rate constants k_2_ and the concentrations in number of moles of solute per mass unit (or surface unit) of adsorbent N. The (N, h) pair showed an increasing linear regression, while the (N, k_2_) pair displayed a decreasing linear regression. The increase in the h values can be attributed to the increase in the driving force, characterized by the mass transfer coefficient k_f_. Under the experimental conditions of this study, the empirical correlation relationships were:

(34) $$h\;=\;1.518\;\times \;10^{5}N,{\;R}^{2}\;=\;0.99$$

(35) $$k_{2}\;=\;-4.52\;\times \;10^{5}N\;+\;3.99,{\;R}^{2}\;=\;0.99$$

This clearly shows that k_2_ is only an apparent constant which depends on the operating conditions, in particular on the concentration of solids. The adsorption rate constant of the pseudo-second-order kinetics for Norit activated carbon was found to be 3.99 g mg^−1^ min^−1^.

The adsorption progress parameter k_2_q_e_, located between 0.6 and 1.03 min^−1^, clearly showed that the adsorption process was fast, especially for CIP. During the time interval studied, the inverse of this parameter corresponded to the half-life of the adsorption process: 1.45 min for SMX, 1.11 min for CBZ and 0.97 min for CIP. Additionally, 50% of the solute was adsorbed between 1 and 1.5 min and the rest was adsorbed over a longer time period. The PSO model thus showed that adsorption was governed by two different rates: a first, fast rate for a short adsorption time followed by a second, slower rate.

In order to take into account all the experimental data, a kinetic model that included the discontinuities produced during the sorption of each solute was required. Following meticulous examination, and given the shape of the overall kinetic curves of CBZ and SMX, it appeared essential to use a sigmoidal function. Therefore, we put forward empirical functions established from the combination of PFO or PSO with a sigmoid (PFO/Sigmoid and PSO/Sigmoid), bearing in mind that the PFO and PSO models were used as a prior step for time intervals below the first discontinuity. Given the many discontinuities observed on the kinetic curve for CIP, these two functions could not be used. A double exponential model formed by combining two first-order reactions proved more appropriate to describe the sorption kinetics of CIP.

For SMX, PSO/Sigmoid showed excellent prediction (R^2^ = 1 and ∆q = 0.75%). However, for CBZ, the two models were roughly equal, with the balance tipping slightly toward the PFO/Sigmoid model (R^2^ = 0.99 and ∆q = 2.58%) (Figure 9B). With these two models, the rate constants (k_1_ and k_2_) were slightly higher than those obtained for the first period with a single model. This slight difference was due to the fitting of the overall model imposed by the sigmoid. After the first discontinuity, sorption q_e,sig_ only represented 35% for CBZ and 11.5% for SMX.

There was a relatively faster sorption rate below the first inflection point and a relatively slower rate after it. These discontinuities indicated a change in environment with a variety of host site types as well as heterogeneity in the surface and volume of the porous compartments of the activated carbon. 

In the case of CIP, agreement between the double exponential prediction model and the experimental data was globally acceptable (∆q = 3.6%,Figure 9C). This model showed that these kinetics were governed by two different adsorption rates, one fast and the second slower. The fast adsorption rate constant nevertheless remained higher than that obtained with PFO for the first period between 0 and 6 min of 37.4%. The significant gaps between the predicted data and the experimental data in relation to the discontinuities clearly showed that this model is only applicable when the amplitudes of these discontinuities are low—for instance in the case of SMX. However, some doubt remains over the distribution of the quantities adsorbed between the fast phase and the slow phase (Table 6). For SMX, the two quantities q_e,r_ and q_e,s_ were close, which contradicted the distribution predicted by the PSO/Sigmoid model. The results of the overall kinetic modelling of CIP with the double exponential model should therefore be taken with considerable caution.

Macroscopically, sorption can be divided into 3 steps: the fast first step involves external diffusion, intraparticle diffusion and adsorption; the slow second step comprises a combination of intraparticle diffusion and adsorption; the final step leads to adsorption equilibrium.

#### 2.3.9. Possible Adsorption Mechanisms

In tap water, pH 8.1 0.1, CBZ is totally neutral, CIP is 1% neutral (CIP0), 85% Zwitterionic (CIP) and 14% anionic (CIP-), SMX is 100% anionic (SMX-) and Norit SA Super activated carbon is anionic. The negative charge of the activated carbon results from the presence of carboxylic (Ar-COO-), phenolic (Ar-O-) and carbonyl (Ar-C=O-) groups. These functional groups favor the formation of hydrogen bonds and complexation, as well as electrostatic interactions (+/−) between this activated carbon and these 3 adsorbates. Moreover, the adsorption of these organic pollutants is strongly favored by dispersion interactions between the π electrons of the aromatic ring and the π electrons of graphene layers (π-π interactions) [62,63]. In activated carbon, the presence of basic groups leads to an increase in the density of delocalized π electrons in the graphene layers and thus, an increase in the carbon’s adsorption potential, while the acidic groups accentuate the local density of π electrons in the graphene layers [63,64]. The effects of water can also be significant in the adsorption mechanisms. Water molecules are likely to be adsorbed onto surface oxygen groups by hydrogen bonding, and in turn become adsorption centers by means of hydrogen bonding. Due to their chemical and electronic structure, these 3 micropollutants can form hydrogen bonds with the activated carbon.

Through its composition and structure, CBZ is able to form π-π stacking interactions between aromatic rings and the graphite plane of the activated carbon [65,66], and in particular the hydrogen bonds. CBZ’s amine group also reacts with the activated carbon’s other functional groups, which contain oxygen such as carbonyl, quinone and phenol (NH…O or NH…N). Furthermore, the addition of CH-π bonds (CBZ-ACP) can prove non-negligible [67]. Given these different possibilities, CBZ can be oriented in different directions around the sheets of activated carbon and inside the porous cavities.

At tap water pH, CIP is predominantly zwitterionic (CIP^±^) with a positively charged amine group in the piperazine ring, and negatively charged carboxyl group. This polarity can induce both electrostatic attraction and repulsion. The anionic CIP^-^ formed due to the loss of a proton from the carboxyl group suggests electrostatic repulsion. Both the zwitterionic and anionic forms are capable of forming complexes with the Ca^2+^, Mg^+^, Na^+^ and K^+^ cations present in tap water [68] and then adsorbing by bridging onto the activated carbon. Additionally, direct adsorption of the zwitterionic form can occur via a columbic attraction between the cationic amine group and the negative sites of the activated carbon. The adsorption of the different forms of CIP to activated carbon can result from electrostatic interactions, π-π coupling [69], hydrogen bonding, or bridging between the negative pole of CIP and the oxygen of the carbon via a cation present in the tap water.

SMX^-^ is able to initiate (i) π-π dispersion interactions and (ii) electrostatic interactions by bridging [70], (iii) the amine group of SMX^-^ can also react with the functional groups of the activated carbon containing oxygen such as carbonyl and phenol, and finally, (iv) the presence of hydrogen bonds is not impossible. As for CIP, the presence of metal cations in low concentrations in tap water can be at the origin of electrostatic interactions between the activated carbon and SMX^-^ by producing bond bridges: SMX^-^/Cation/ArO^-^ and SMX^-^/Cation/ArCOO^-^. The interaction force of these cations with the negative functional groups increases as the hydrated radius of the cation decreases and its polarizability increases, its charge increases, and as its ability to hydrate decreases. 

## 3. Materials and Methods

### 3.1. Materials 

All reagents and solvents used were of analytical grade. Finely dispersed powders of carbamazepine, ciprofloxacin and sulfamethoxazole of high purity (>99.9%) were purchased from Sigma Chemical. Norit SA Super (ACP) powdered activated carbon obtained from peat was supplied by JACOBI CARBONS (Cabot Norit Activated Carbon, Amersfoort, The Netherlands). CBZ, CIP, SMX and ACP were used as received.

### 3.2. Methods

The particle size distribution of ACP was obtained by helium-neon laser diffraction (632.8 nm) using the Malvern Mastersizer S particle size analyzer (Malvern Panalytical, Malvern, UK) by applying the Fraunhofer optical model. The specific surface areas, the pore volumes and the slit widths less than 50 nm were determined at 77 K from nitrogen adsorption/desorption isotherms using a Micromeritics ASAP 2050 discontinuous volumetry sorptometer (Micromeritics Instrument Corp., Norcross, GA, USA). Prior to analysis, ACP was dried and degassed at 383 K until a residual vacuum of less than 0.02 mbar using the Micromeritics AccuPyc 1330 degassing ramp (Micromeritics Instrument Corp., Norcross, GA, USA). The BET method was used to determine the specific surface area. The t-plot method was used to assess the main characteristics of the micropores from the desorption isotherms. In addition to textural analysis, micropores smaller than 2 nm were characterized by CO_2_ adsorption at 273 K according to the Dubinin–Astakhove method [71]. The molecular cross-sectional areas of N_2_ and CO_2_ are 0.162 nm^2^ and 0.187 nm^2^ respectively. The point of zero charge (PZC) was determined using the Mular–Roberts [72] method, known as the pH drift method. The isoelectric point (IEP; the pH value where the electrophoretic mobility equals zero) were performed using the Malvern Zetasizer Nano-ZS (Malvern Instruments Ltd.). The activated carbon surface functional groups were identified and quantified by acid–base titration following the Boehm method [44,45,46]. Their determination was performed with NaHCO_3_ for carboxylic acid functions, with Na_2_CO_3_ for lactone and carboxylic acid functions, with NaOH for phenol, lactone and carboxylic acid functions and with HCl for basic functions. Excess base was back titrated with an HCl solution. 

Kinetics adsorption were determined with batch experiments at 20 ± 1 °C. Experiments were conducted in drinking water. This water had a pH of 8.1 ± 0.1, a conductivity of 352 μS/cm at 25 °C, an ionic force of 5.4 ± 0.4 mmol/L and a chemical composition with a dissolved salt concentration of 259 ± 3 mg/L with 3.35 meq/L for cations and 3.51 meq/L for anions. The quantification of pharmaceutical compounds was performed by liquid chromatography coupled to mass spectrometry. The limits of detection and quantification are 0.7 ng/L and 2 ng/L. The solutions were prepared by dilution in amber high-density polyethylene bottles at ambient temperature.

## 4. Conclusions

The main characteristics necessary to evaluate and predict the behavior of CBZ, CIP and SMX in suspension were clearly formulated. A new empirical relationship of the solubility of each pollutant was proposed. The activated carbon used proves very effective for the nonspecific treatment of very small quantities of these contaminants. A concentration of 2 µg/L can be completely eliminated by less than 10 mg/L of activated carbon.

The sorption process of these 3 molecules is governed by external diffusion, intraparticle diffusion and relatively rapid chemisorption during the first few minutes of contact between the adsorbent and the adsorbate. Using the Homogeneous Surface Diffusion Model (HSDM), the Weber–Morris model and a method put forward in this study to address external diffusion, the mass transfer coefficients, surface diffusion coefficients, effective diffusion coefficients, pore diffusion coefficients and the solute concentration at the outer surface of the adsorbent were easily determined. At the same temperature and in similar hydrodynamic conditions, CIP reached the liquid film around the particles of activated carbon quicker than CBZ and SMX. However, inside the pores, CIP had a low surface diffusivity compared to the two other molecules. These differences can be explained by the geometric dimensions, form factors and electrochemical volumes of these molecules. Based on a statistical analysis, it was shown that the surface concentration, the mass transfer coefficients and the diffusion coefficients were strongly correlated to the molecular geometry, namely the cross-sectional area formed by the arithmetical product of the length by the height of each molecule.

In the presence of major observable discontinuities in kinetic curves, traditional kinetic models are not suitable for modeling the experimental data set. Pseudo-first and second order models can be used to fit only the data below the first discontinuity. In order to take into account all the experimental data for the kinetics of CBZ and SMX, the use of a PSO/Sigmoid kinetic model, proposed in this research, was required. The double exponential model was moderately suited to the study of CIP kinetics.

In terms of future prospects, it would be useful to extend the approach applied in this article to other adsorbents and pharmaceutical pollutants in order to generalize the correlations found and the applicability of the new kinetic model proposed here.

## Figures and Tables

**Table 1:** Intrinsic physicochemical properties of CBZ, CIP and SMX.

Parameter	Carbamazepine	Ciprofloxacin	Sulfamethoxazole
CAS number	298-46-4	85721-33-1	723-46-6
Molecular formula	C_15_H_12_N_2_O	C_17_H_18_FN_3_O_3_	C_10_H_11_N_3_O_3_S
Molecular weight(g mol^−1^)	236.27	331.34	253.28
BCS (class drug)	II	IV	IV
Melting point (°C)	175	268	167
Density (g cm^−3^)	1.343	1.453	1.462
Crystal system (anhydrous form)	P-monoclinic [28] a = 7.537 Å, b = 11.156 Å, c = 13.912 Å, α = γ = 90°, β = 92.862° & Z = 4	Triclinic [29] a = 8.062 Å, b = 9.730 Å, c = 10.321 Å, α = 99.927°, β = 104.541°, γ = 98.073 & Z = 2	Monoclinic [30] a = 24.719 Å, b = 7.204 Å, c = 14.663 Å, α = γ = 90°, β = 118.217° & Z = 8
Molecular width (nm)	0.507 [31]	0.82 [32]	0.526 [31]
Molecular height (nm)	0.529 [31]	0.25 [32]	0.587 [31]
Molecular length (nm)	0.891 [31]	1.31 [32]	1.031 [31]
Acid dissociation constant	pK_a1_≈1; pK_a2_ = 13.9 [33]	pK_a1_ = 6.0; pK_a2_ = 8.8 [32]	pK_a1_ = 1.8; pK_a2_ = 5.6 [34]
Log P^IEP^	2.25 [35]	−1.08 [36]	0.89 [31]
Log D_8_ (*)	2.25	−1.15	−1.54
Hydrosolubility (solute in MQ-water), S (mg L^−1^), T(°C) < 60 (*)	S = P_1_T + P_2_T^P3^ P_1_ = 4.35, P_2_ = 6.09 × 10^−5^, P_3_ = 3.88	S = P_1_T + P_2_T^P3^ P_1_ = 3.04, P_2_ = 7.64 × 10^−5^, P_3_ = 3.63	S = P_1_T + P_2_T^P3^ P_1_ = 14.81, P_2_ = 4.66 × 10^−6^, P_3_ = 4.43
Acceptable daily intakes for human (µg kg^−1^ day^−1^) [37]	11	2	24

BCS: Biopharmaceutics Classification System of drug. K_a_: acid dissociation constant (expressed as -Log K_a_ = pK_a_). Log D_8_: Logarithm of the octanol–water distribution coefficient at pH 8. Log P^IEP^: Logarithm of the octanol–water partition coefficient calculated at the isoelectric point. Z: Molecules per unit cell. * Calculated values and formulas proposed in this study.

**Table 2:** Main characteristics of ACP Norit SA Super.

d_a_	IEP	PZC	SS_BET_	SS_µP-t_	SS_mP-t_	SS_MP-t_	L_µ-t_	V_µP-t_	V_mP-t_	V_MP-t_	SS_µP-DA_*	V_µP-DA_ *
µm			m^2^ g^−1^	m^2^ g^−1^	m^2^ g^−1^	m^2^ g^−1^	nm	cm^3^ g^−1^	cm^3^ g^−1^	cm^3^ g^−1^	m^2^ g^−1^	cm^3^ g^−1^
24	3.6	6.8	957	695	203	60	1.75	0.30	0.17	0.10	711	0.31

d_a_: average diameter. IEP: isoelectric point. PZC: point of zero charge. t: t-plot method. DA: Dubinin–Astakhov method. *: Analysis done with CO_2_. SS: specific area. SS_BET_: BET-specific surface area. SS_µP_: equivalent specific surface area of micropores. SS_mP_: equivalent specific surface area of mesopores. SS_MP_: equivalent specific surface area of macropores. L_µ-t_: mean equivalent pore width (t-Plot). V_µP_: specific micropore volume. V_mP_: specific mesopore volume. V_MP_: specific macropore volume.

**Table 3:** Functional groups on activated carbon Norit SA Super by Boehm analysis.

Functional Group	Density µmol m^−2^	Number of Sites per nm^2^
Carboxylic	0.28	0.17
Carbonyl	0.46	0.28
Anhydride	0.17	0.10
Lactone	0.06	0.03
Phenol	0.53	0.32
Total electron donor	0.96	
Total electron acceptor	0.80	

**Table 4:** Diffusional parameters.

Pollutant	m_p_/m_ACP_ µg mg^−1^	k_f_ cm min^−1^	C_S_ µg mg^−1^	D_S_ cm^2^ min^−1^	D_e_ cm^2^ min^−1^
SMX	0.2	0.20	0.09	12.3 10^−10^	3.1 10^−8^
CBZ	0.4	0.51	0.11	3.4 10^−10^	1.7 10^−8^
CIP	0.8	1.14	0.14	0.9 10^−10^	1.1 10^−8^

m_p_ is the mass of solute; m_ACP_ is the mass of ACP; k**_f_** is the external mass transfer coefficient (at ±0.05); C_S_ is the solid-liquid interface (at ±0.01), DS is the surface diffusion coefficient (at ±0.1 10^−10^); De is the effective diffusion coefficient (at ±0.1 10^−8^).

**Table 5:** (**a**) Correlation matrix (Pearson). (**b**) *p*-values (Pearson). (**c**) Coefficients of determination (Pearson).

(**a**)															
**Variables**	**MW**	**Log1/MW**	**LxW**	**LxH**	**WxH**	**LxWxH**	**k_f_**	**C_s_**	**D_e_**	**D_s_**	**Logk_f_**	**LogC_S_**	**LogD_e_**	**LogD_S_**	**q_e,exp_**
MW	**1**														
Log1/MW	**1.000**	**1**													
LxW	**0.999**	**0.998**	**1**												
LxH	−0.783	−0.766	−0.803	**1**											
WxH	−0.844	−0.829	−0.861	0.995	**1**										
LxWxH	0.023	0.050	−0.011	0.604	0.518	**1**									
k_f_	0.879	0.865	0.894	−0.985	**−0.998**	−0.457	**1**								
C_S_	0.820	0.804	0.839	**−0.998**	**−0.999**	−0.553	0.994	**1**							
D_e_	−0.625	−0.603	−0.651	0.975	0.946	0.766	−0.922	−0.959	**1**						
D_s_	−0.539	−0.516	−0.567	0.946	0.907	0.830	−0.876	−0.924	0.994	**1**					
Logk_f_	0.741	0.723	0.763	**−0.998**	−0.986	−0.654	0.972	0.992	−0.987	−0.965	**1**				
LogC_s_	0.782	0.764	0.802	**−1.000**	−0.994	−0.606	0.985	**0.998**	−0.975	−0.947	**0.998**	**1**			
LogD_e_	−0.731	−0.712	−0.753	0.997	0.983	0.666	−0.968	−0.990	0.990	0.969	**−1.000**	**−0.997**	**1**		
LogD_s_	−0.781	−0.763	−0.801	**1.000**	0.994	0.607	−0.984	**−0.998**	0.976	0.947	**−0.998**	**−1.000**	**0.997**	**1**	
q_e,exp_	0.778	0.760	0.799	**−1.000**	−0.994	−0.611	0.984	**0.998**	−0.977	−0.949	**0.998**	**1.000**	**−0.997**	**−1.000**	**1**
(**b**)															
**Variables**	**MW**	**Log1/MW**	**LxW**	**LxH**	**WxH**	**LxWxH**	**k_f_**	**C_s_**	**D_e_**	**D_s_**	**Logk_f_**	**LogC_s_**	**LogD_e_**	**LogD_s_**	**q_e,exp_**
MW	**0**														
Log1/MW	**0.017**	**0**													
LxW	**0.021**	**0.039**	**0**												
LxH	0.427	0.445	0.406	**0**											
WxH	0.361	0.378	0.340	0.067	**0**										
LxWxH	0.986	0.968	0.993	0.587	0.654	**0**									
k_f_	0.317	0.334	0.296	0.111	**0.044**	0.698	**0**								
C_s_	0.388	0.405	0.366	**0.040**	**0.027**	0.627	0.071	**0**							
D_e_	0.570	0.588	0.549	0.143	0.210	0.444	0.254	0.183	**0**						
D_s_	0.637	0.655	0.616	0.210	0.276	0.377	0.320	0.250	0.067	**0**					
Logk_f_	0.469	0.486	0.447	**0.041**	0.108	0.546	0.152	0.081	0.102	0.169	**0**				
LogC_s_	0.429	0.446	0.407	**0.001**	0.068	0.586	0.112	**0.041**	0.142	0.209	**0.040**	**0**			
LogD_e_	0.478	0.496	0.457	0.051	0.117	0.536	0.161	0.090	0.092	0.159	**0.010**	**0.049**	**0**		
LogD_s_	0.430	0.447	0.408	**0.002**	0.069	0.585	0.113	**0.042**	0.141	0.208	**0.039**	**0.001**	**0.049**	**0**	
q_e,exp_	0.433	0.450	0.411	**0.005**	0.072	0.582	0.116	**0.045**	0.138	0.205	**0.036**	**0.004**	**0.046**	**0.003**	**0**
(**c**)															
**Variables**	**MW**	**Log1/MW**	**LxW**	**LxH**	**WxH**	**LxWxH**	**k_f_**	**C_s_**	**D_e_**	**D_s_**	**Logk_f_**	**LogC_s_**	**LogD_e_**	**LogD_s_**	**q_e,exp_**
MW	**1**														
Log1/MW	0.999	**1**													
LxW	0.999	0.996	**1**												
LxH	0.613	0.586	0.645	**1**											
WxH	0.712	0.687	0.742	0.989	**1**										
LxWxH	0.001	0.002	0.000	0.365	0.268	**1**									
k_f_	0.772	0.749	0.800	0.970	0.995	0.209	**1**								
C_S_	0.673	0.647	0.704	0.996	0.998	0.306	0.988	**1**							
D_e_	0.390	0.364	0.423	0.950	0.896	0.587	0.850	0.920	**1**						
D_s_	0.291	0.267	0.322	0.895	0.823	0.688	0.767	0.854	0.989	**1**					
Logk_f_	0.549	0.522	0.583	0.996	0.972	0.428	0.944	0.984	0.975	0.931	**1**				
LogC_s_	0.611	0.584	0.643	1.000	0.989	0.367	0.969	0.996	0.951	0.897	0.996	**1**			
LogD_e_	0.534	0.507	0.568	0.994	0.966	0.443	0.937	0.980	0.979	0.939	1.000	0.994	**1**		
LogD_s_	0.610	0.583	0.642	1.000	0.988	0.368	0.969	0.996	0.952	0.897	0.996	1.000	0.994	**1**	
q_e,exp_	0.605	0.578	0.638	1.000	0.987	0.373	0.967	0.995	0.954	0.900	0.997	1.000	0.995	1.000	**1**

Values in bold are different from 0 with a significance level α = 0.05.

**Table 6:** Adsorption kinetics parameters.

						**Pseudo-First-Order(PFO)**				**Pseudo-Second-Order (PSO)**					
**Pollutant**	**m_p_/m_ACP_**	**N**	**Interval**	**k_f_**	**q_e,exp_**	**q_e,calc_**	**k_1_**	**R^2^**	**∆q**	**q_e,calc_**	**h**	**k_2_**	**k_2_q_e_**	**R^2^**	**∆q**
	mg g^−1^	mol g^−1^	time (min)	cm min^−1^	mg g^−1^	mg g^−1^	min^−1^		%	mg g^−1^	mg g^−1^ min^−1^	g mg^−1^ min^−1^	min^−1^		%
SMX	0.2	7.90 10^−7^	0–12	0.20	0.166	0.162	0.503	0.994	4.07	0.186	0.126	3.653	0.680	0.999	0.76
CBZ	0.4	1.69 10^−6^	0–6	0.50	0.235	0.236	0.786	0.998	2.85	0.283	0.256	3.190	0.904	0.998	2.26
CIP	0.8	2.41 10^-6^	0–6	1.12	0.307	0.299	0.864	0.996	3.93	0.353	0.365	2.924	1.033	0.999	2.32
				**PFO/Sigmoid**					**PSO/Sigmoid**						
**Pollutant**	**m_p_/m_ACP_**	**Interval**	**q_e,exp_**	**q_e,calc_**	**q_e,sig_**	**k_1_**	**R^2^**	**∆q**	**q_e,calc_**	**q_e,sig_**	**h**	**k_2_**	**k_2_q_e_**	**R^2^**	**∆q**
	mg g^−1^	time (min)	mg g^−1^	mg g^−1^	mg g^−1^	min^−1^		%	mg g^−1^	mg g^−1^	mg g^−1^ min^−1^	g mg^−1^ min^−1^	min^−1^		%
SMX	0.2	0–30	0.198	0.200	0.049	0.601	0.999	2.05	0.208	0.023	0.128	3.724	0.690	1.000	0.75
CBZ	0.4	0–20	0.398	0.403	0.174	0.832	0.999	2.58	0.413	0.139	0.273	3.660	1.000	0.999	2.69
**Double-Exponential Model**															
**Pollutant**	**m_p_/m_ACP_**	**Interval**		**q_e,exp_**	**q_0_**	**q_e,r_**	**q_e,s_**	**q_e1_ + q_e2_**	**k_s_**			**k_r_**		**R^2^**	**∆q**
	mg g^−1^	time (min)		mg g^−1^	mg g^−1^	mg g^−1^	mg g^−1^	mg g^−1^	min^−1^			min^−1^			%
SMX	0.2	0–30		0.198	−8.8 10^−4^	0.100	0.119	0.220	0.061			0.770		0.996	2.3
CIP	0.8	0–80		0.798	−2.7 10^−4^	0.630	0.216	0.847	0.029			1.381		0.995	3.6

m_p_: mass of solute; m_ACP_: mass of ACP; N: number of moles of solute per mass unit.

## Data Availability

Not applicable.
